# Development, production and evaluation of 2-dimensional transfer tattoos to simulate skin conditions in health professions education

**DOI:** 10.1186/s12909-021-02763-z

**Published:** 2021-06-21

**Authors:** Daniel Bauer, Andrea C. Lörwald, Sandra Wüst, Helmut Beltraminelli, Miria Germano, Adrian Michel, Kai P. Schnabel

**Affiliations:** 1 Institute for Medical Education; Faculty of Medicine, University of Bern, Mittelstrasse 43, 3012 Bern, Switzerland; 2 Department of Dermatology, Inselspital, Bern, Switzerland

**Keywords:** Medical moulage, Simulation engagement, Clinical skills, OSCE, Standardized patient

## Abstract

**Background:**

Moulages can greatly extend the possibilities of simulation in teaching and assessment. Since moulages that fit an educator’s exact needs are often unavailable commercially, this paper explains how 2-dimensional transfer tattoos can be independently developed, produced, and evaluated.

**Methods:**

From representative photographs of the specific skin condition an analogue copy of the pathological finding is drawn. Once validated by the medical expert, it can be digitized by scanning and processed using graphics software. The final digital image file is printed onto transfer paper. Once applied and fixed onto the intended wearer, usually a simulated patient, its authenticity can be confirmed, and further transfer tattoos can be produced.

**Results:**

Using this moulage technique we produced 10 different 2-dimensional transfer tattoos to date, including hematoma, Janeway lesions and splinter nails. These moulages are used in clinical skills training, formative and high-stakes summative assessment in undergraduate medical and nursing programs.

**Conclusions:**

By sharing our development process for 2-dimensional transfer tattoos, health profession educators can produce their own that best fit their local educational needs. Due to their high authenticity and standardization, 2-dimensional transfer tattoos are ideal for use in high-stakes assessment.

**Supplementary Information:**

The online version contains supplementary material available at 10.1186/s12909-021-02763-z.

## Background

Since its inception in the 1960s, simulated patient (SP) methodology has spread around the world and is now widely accepted in simulation-based teaching and assessment in health professions education. SPs can portray many pathological conditions [1]. For those non-portrayable, workarounds include the use of simulators (e.g., delivery), part-task trainers (e.g., rectal exam), or material presented as video (e.g., seizures), audio (e.g., heart murmurs) or photographs (e.g., rashes). However, these all constitute media breaks which can negatively influence the learners’ or examinees’ simulation engagement [2] and threaten the simulation’s validity argument. One method to remedy this is the use of modern medical moulage. Moulages allow the depiction of visual findings directly on the SP, rendering the delivery of photographs by the simulation facilitator or clinical examiner redundant. The range of findings that can be displayed with moulages encompasses traumatological [3–6], infectious diseases and medical conditions [7–10], and dermatological findings [11–16]. While there are examples where moulages were mounted onto a mannequin [17–19], they are most commonly applied directly onto SPs [20–22]. Educational scenarios in which moulage are used are diverse. Garg et al. describe the use of moulage in a dermatology course and report a positive effect on long-term learning outcomes [12]. Other uses include the use of moulage in the assessment of clinical skills, e.g., detecting [21] and managing skin lesions [23]. Beyond examination skills, moulage have also been employed to stimulate empathy in learners [16].

While modern medical moulage employs technologies used in the movie industry, its use in health professions education demands an additional set of criteria. While most moviegoers will not be put off by minor medical inconsistencies, the intended users of moulage in medical simulation usually have at least some expertise, also considering inconspicuous details during the simulation, while building their diagnostic and therapeutic plan. Correct tactile information adds another criterion relevant in simulations but not movies. Beyond these authenticity issues, the use of moulages in medical assessment requires the best possible produce quality and high levels of standardization ensuring a level playing field for examinees and as not to compromise the justiciability of the exam. In the following we provide a short overview of different approaches to medical moulages in simulation.

### 2-dimensional moulage techniques

- Special effects makeup, based on grease, water or alcohol soluble colours, allows for relatively quick and cheap application of 2-dimensional findings onto an SP. They can be used to cover large areas of the SP’s body including hairy areas and near the eyes (water and grease-based colours) and can be edited easily if necessary. Their degree of standardization depends primarily on the makeup artist’s expertise, the photo template used and desired granularity of detail. The indication for special effects makeup includes scenarios where the findings depicted serve for quick diagnoses and decisions, e.g. in mass casualty triage, or as visual cues. They can usually last for at least a day but do not convey spatial or tactile information.
- 2-dimensional transfer tattoos are printed on decal paper and have a higher potential for standardization. Development requires a good photographic template and some artistic and digital editing skills. Production and application are quick and easy [13]. They can be used to cover large, preferably flat areas of the SP’s body including finger- and toenails, but usually excluding overly hairy areas. The quality depends on the base image and printing process. Once mounted, additional editing is limited. Indications include medical conditions of a 2-dimensional nature (e.g., some rashes). Can last for several days but convey neither spatial nor tactile information.

### 3-dimensional moulage techniques

3-dimensional moulages open a new range of findings that can be simulated, as they include spatial and sometimes correct tactile information.

- 3-dimensional moulage can be hand-modelled directly onto the SP or simulator, often using wax, silicone, or gelatine materials (gelatine-based moulage can be modelled into hairy areas including the scalp). The development phase includes obtaining good photographs as templates. The application takes time and is highly dependent on the makeup artist’s skill and experience. Once applied, these moulages can be further edited as required. Standardization depends heavily on the desired granularity of details and again on the makeup artist. Hand-modelled moulages are a relevant choice for scenarios with lower requirements for standardization and detail, e.g., triage exercises, for budget-constrained settings and for moulage requested at short notice. These moulages can hold for several hours, limited mostly by their adherence to the SP.
- 3-dimensional silicone appliances are quite sturdy and allow reutilization. Development and production are quite elaborate, but application is quick and easy. Once mounted, the degree of further editing is limited. Their sturdiness often comes at the price of increased thickness, so the transition into the SP’s own skin often remains clearly visible [24]. Their size can be considerable but tend to stick out as artificial with increasing size. This is of less importance in scenarios where the edges of the moulage would be covered anyway (e.g., in some wound care tasks) or when the moulage is not crucial for reaching a diagnosis but serves to demonstrate a clinical procedure. Within these limitations, their high standardization and commercial availability makes them suitable for certain assessment settings. Tactile authenticity is seldomly achieved.
- A variation of 3-dimensional moulages, prepared in advance, makes use of movie makeup artistry to produce thin-edged *Probondo* transfer tattoos, at the cost of being single-use. They have the same advantages as the thicker versions described. Like printed tattoos, they offer high levels of standardization, however at the cost of an elaborate development and production process. Their authenticity depends largely on the makeup artist’s expertise. A specialist’s suggestions are crucial regarding peculiar morphological parameters to fine-tune colour nuances, distribution, size, and shape of the lesion(s). Since these moulages blend well into the SP’s own skin, they can be used in scenarios where they should not be too obvious to spot and require learners or examinees to uncover and interpret them, before building their diagnostic and therapeutic plan. Can be applied quickly and easily and last for a whole day, especially if attached with sealer. Once mounted, the degree of further editing is limited. Can convey tactile information.

Moulages offer the possibility to integrate a myriad of pathologies into simulation visually. However, the range of commercially available moulages is narrow and their authenticity not necessarily up to every educator’s individual needs. In order to independently prepare 2-dimensional transfer tattoos for their own simulation needs, educators have to overcome some obstacles. These include achieving a medically correct visualisation of the pathology and then affixing that visualisation onto the wearer (SP or mannequin), guaranteeing a smooth visual transition into the SP’s own skin. For this, relevant competencies beyond medical expertise are needed and include painting skills, digital image editing, and knowledge of transfer materials and techniques. Since these are skills not all medical educators have, our aim is show how to approach the preparation of medical transfer tattoos with little prior knowledge and how to how to integrate these skills. By doing so we will enable people with little experience to prepare their own 2-dimensional moulages using easily available materials.

## Methods

### Preparing an analogue template

The process begins with the provision of representative, high-resolution photographs of the specific skin condition by a medical expert. From this, the artist draws an analogue copy of the pathological finding, i.e., everything *but* the healthy skin. This is done with alcohol or water-soluble colours on a semi-transparent parchment-like paper. A normalized colour fan acts as inexpensive and handy common standard between artist and medical expert when discussing colour nuances. Using a common industrial standard allows for reliable identification of colour values, conversion into other colour systems, and direct comparison with the physical product. Minor retouching and corrections can usually be realized digitally later.

### Digitizing the data

Once the medical expert confirms the correctness of the drawing, it is fixed onto a blank sheet of regular white office paper using adhesive tape for scanning. The scan engine of a regular office printer delivers useable results (full colour text/photo scan, 600 dpi, pdf file format).

Once digitized, the image is processed using Adobe Photoshop. First, the yellowish tint of the parchment-like paper is colour-corrected using the graduation curve adjustment layer. Colours are enhanced using the vibrance adjustment layer. Regular proofs are printed while keeping print settings unchanged to show if further colour adjustment is necessary. The original photographs and the expert-validated drawing act as optical references.^1^

### Manipulating the template

In some cases (e.g., when designing a rash) one might decide to prepare just a section of the rash and digitally increase its size. To achieve this, the digital template produced to begin with can be flipped, inverted, and otherwise transformed multiple times, with the parts then spliced together to increase the size of the finding which will be printed later, avoiding a tiled appearance. Prominent parts of the condition that could give away this manipulation can be excluded from this process and later re-set into the picture once its desired size is achieved.

With skin conditions where the orientation of the finding is of importance, the print needs to be mirror-inverted, as the transfer paper is flipped over when applying the tattoo to the SP’s skin.

If applied to the SP’s skin, every colour point printed on it will, as a rule, subtly darken the underlying skin. Only yellow colour tones can have a brightening effect. The SP’s skin colour thus defines the brightest possible hue of the presented skin condition (subtractive colour mixing), therefore depicting, e.g., dried pus or dandruff is unfeasible.

### Printing and finishing the transfer tattoo

The final digital image file is then printed on transfer paper, using the previous print settings. The prints can be stored in this form until used.

#### Application and Evaluation

**Fig. 1:**
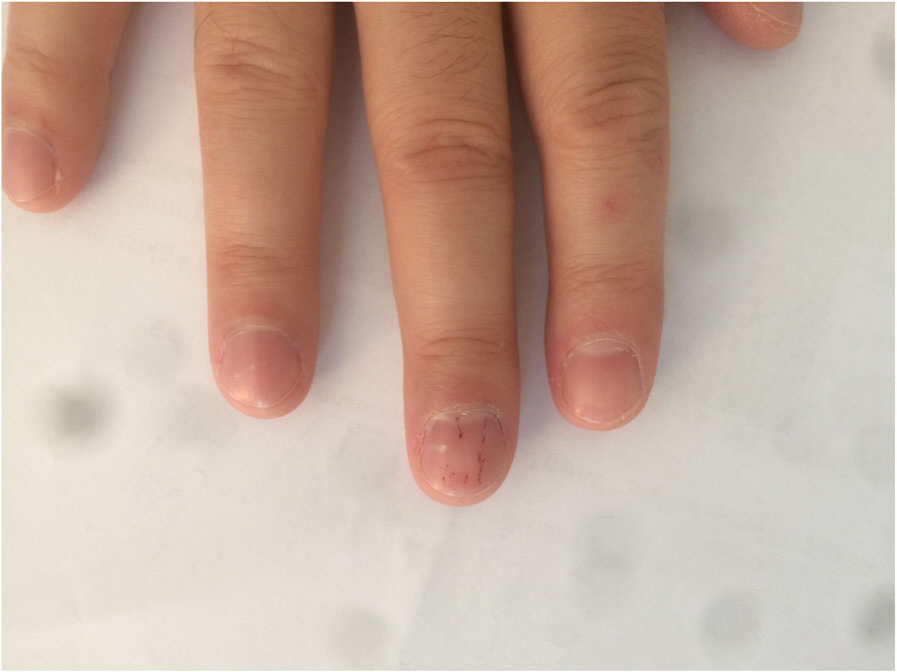
A 2D tattoo prototype of splinter nails on a healthy 40-year-old SP’s middle finger, intended for a case of bacterial endocarditis. The haemorrhages should be less sequential but continuous, parallel to each other as well as the nail’s growth lines. The colouring suggests an old subungual haemorrhage

To determine if the 2D transfer tattoo produced in this manner is authentic, it needs to be fixed onto the intended wearer. For this, the front of the print is sprayed with adhesive spray and left until completely dry. Scissors are used to cut off superfluous sections. It is then placed onto the intended place on the wearer’s body, the paper facing upwards, away from the body (i.e., the print facing the SP’s skin). The paper is then soaked with water until it can be moved sideways, carefully, and easily. Once any remaining water has evaporated, 96% isopropyl-soaked cotton swabs can be used to remove any unwanted residual paste on the edges. Then sealer^2^ is applied gently with a fresh cotton swab, airbrush, or sponge, covering the whole moulage and immediately adjacent areas and left until completely dry.^3^ The sealer fixes the moulage, so it does not stick, collect lint or tear when manipulated.^4^ The applied transfer tattoo can now be judged against prior established criteria or other tools [25]. If unsatisfactory, earlier steps must be revisited. As soon as the product is satisfactory, production can start. Figure 1 depicts an example of a print in need of further editing.

### Removing the moulage

After use, soak cotton pads with makeup remover and gently rub the moulage starting at the edges. Use additional rehydrating skin care as warranted.

### Materials

Table 1 Additional material: paintbrushes, 96% isopropyl, colour fan deck, office quality white paper, office quality adhesive tape, office scissors, cotton swabs, cotton pads. Throughout the production process, maintain safety standards as per manufacturer’s specifications and institutional and national guidelines.

**Table 1:** Components used in the development and production of 2D Transfer Tattoos

***Component***	***Product name***	***Producer***
*Tracing paper*	*High-quality tracing paper 80 g/m2, smooth front and matt back surface*	*AMI Art Material International Warenhandelsgesellschaft mbH, Kaltenkirchen, Germany*
*Alcohol soluble colours*	*PPI Skin Illustrator,* e.g.*, FX Palette*	*Premiere Products Inc., Pacoima, CA, USA*
*Printer/Scanner*	*Aficio Infotec MP C2800*	*Ricoh Company Ltd., Tokio, Japan*
*Colour Calibration*	*Spyder 4 Elite*	*Datacolor AG, Lucerne, Switzerland*
*Image processing hardware*	*iMac (27-in., Late 2013 configuration)*	*Apple Inc., Cupertino, CA, USA*
*Image processing software*	*Photoshop CS5.5*	*Adobe Systems, San Jose, CA, USA*
*Transfer paper*	*Tattoo 2.1 Transfer Paper*	*TheMagicTouch GmbH, Dieburg, Germany*
*Adhesive Spray*	*Golden Phoenix Super Spray Adhesive*	*Baoding OUPU Electronic Science and Technology Co. Ltd, Baoding, China* *gpbodyart.com*
*Makeup remover*	BEN NYE Bond Off!!	Ben Nye Company, Inc., Los Angeles, CA, USA

The process of developing and producing 2D transfer tattoo moulages described above is generic and should work with materials of different producers. For reproducibility reasons, this table declares the precise materials used by the authors

## Results

**Fig. 2:**
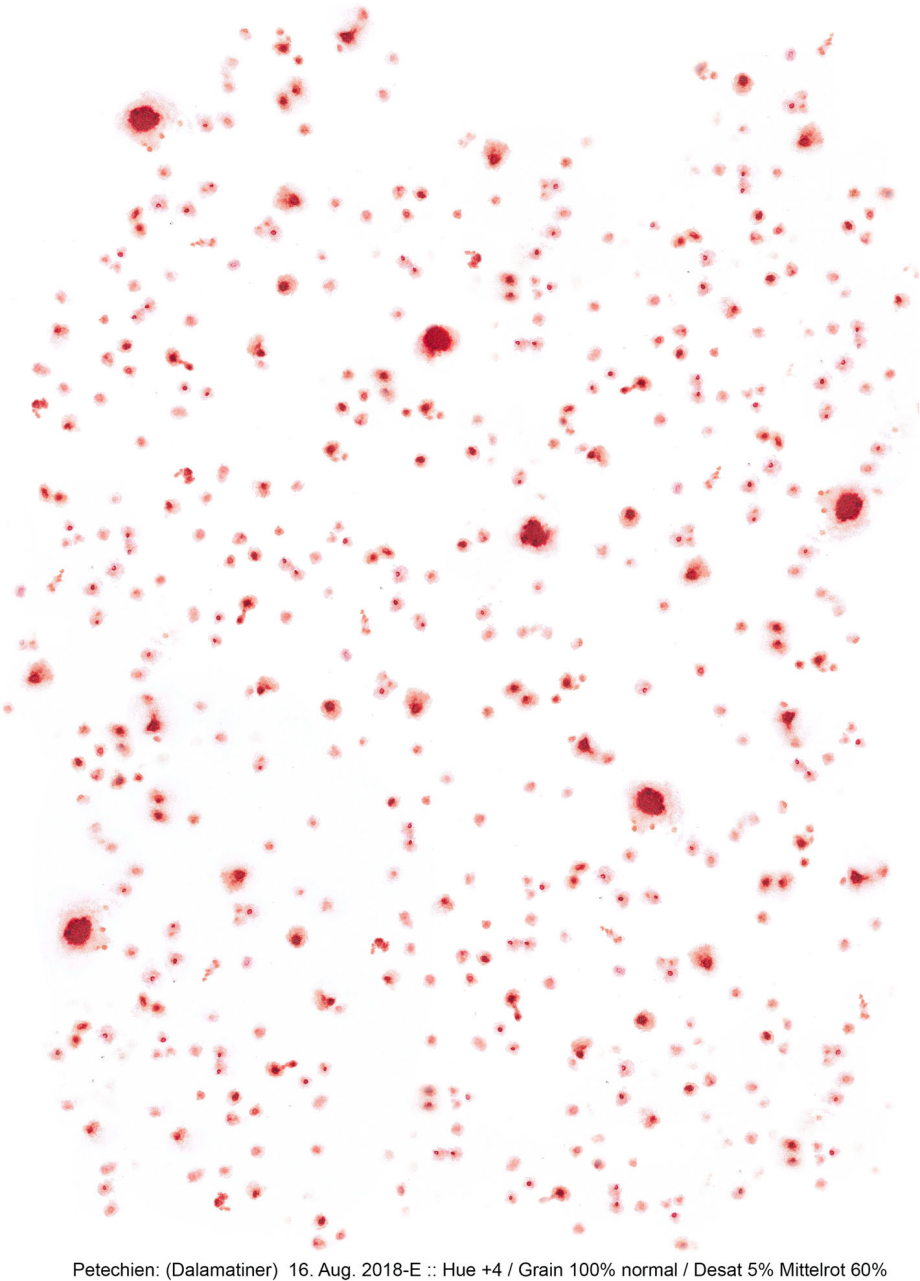
The print template to a case of petechial bleeding in a patient with thrombocytopenia. The image is shrunk in size from 21 by 29.7 cm in the original

**Fig. 3:**
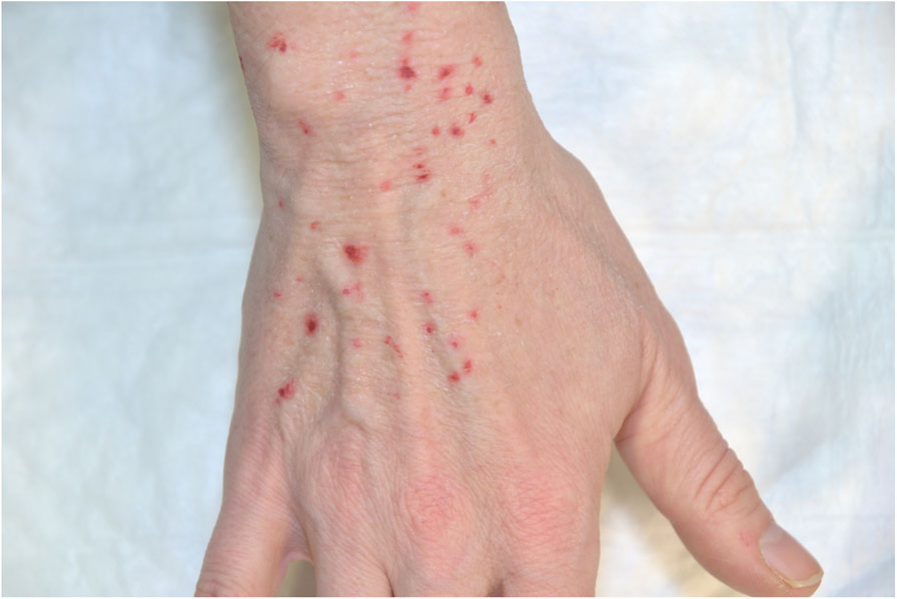
The 2D Tattoo affixed onto an SP. The knuckles and back of the hand were left out, as regular flexing would have broken the print

Using this technique, we were able to produce 10 different 2-dimensional (e.g., hematoma, Janeway lesions, splinter nails) moulages. They are currently used in formative and high-stakes summative assessment (including the Swiss Federal Licensing Examination [26]) as well as clinical skills training in undergraduate medical and nursing programs. Figure 2 depicts an example of a print used in an exam as print template, Fig. 3 shows the same tattoo affixed onto an SP.

To ensure highest quality, we integrated a sequence of control steps. When planning an exam, a meeting with exam stakeholders and the makeup artist is scheduled to determine which cases should and can be realized with moulages, considering the overall exam and available resources and, if possible, showcasing prototypes. After this meeting development and production take place as described. On the morning of the exam day, examiners covering any moulages case are invited to assess the moulages affixed to the SP. Based on the examiners’ feedback, moulages are either approved, or can still get touched up, exchanged with a backup moulage or in extreme cases taken off, reverting to backup photographs. SPs are instructed to reach out to examination support staff should they notice any problem with their moulage (well-prepared moulage usually hold for at least half a day, often longer). In this case, a (moulaged) backup SP takes their place while makeup staff tend to the moulage. SPs are also directed to report to makeup staff during breaks for check-up. Photographic documentation of the moulages further supplements quality control.

Once the exam is over, we collect systematic and casual feedback from the examiners, examinees, and observations from makeup staff regarding the moulages, e.g., durability. SP feedback regarding moulages is collected during the debriefing session. Relevant information regarding the moulages is discussed in a post-exam debriefing meeting and should the need arise, acted upon accordingly.

## Discussion

Moulages can greatly extend the possibilities of simulation but since their dissemination is low and since moulages that fit an educator’s exact needs are often unlikely to be available commercially. This paper describes how we developed, produced, and evaluated 2-dimensional moulages ourselves.

We used our moulages mainly for high-stakes assessment in which authenticity and standardization were deemed of importance. During assessment, examiners, SPs, and makeup staff were key for quality control. After the exams, we obtained additional feedback from examinees and more elaborate feedback from examiners and SPs as basis for future development.

Moulages can and should be evaluated in a more systematic manner, e.g., using the MARS instrument [25]. This tool takes into account not only the anatomical or visual correctness of a moulage (e.g., regarding colour, size, position, shape, likeness to real world) but also whether it fits the scenario (e.g., logical fit) and its effect on the participant (does not distract/confuse the participant).

Development and production of a moulage as described requires some resources (time, materials, a makeup artist, and access to clinical experts). Before even embarking the development of a moulage, the effort and assumed benefit of using moulages should be determined. This requires some knowledge of the educational scenario and the specific case, both in teaching scenarios and assessment. Is the affliction that is to be realized central to the case, relevant for the learner or examinee to correctly reach the diagnosis and build their management plan; would the affliction be missed if it were not visible on the SP; is the affliction scripted in the case as near the eyes, on mucous membranes, on joints that get flexed a lot or hairy parts of the body, which count as relative contraindications for moulages, or could the case be adapted to better fit the simulation with a moulage; will the resources be available on the day required to competently apply the moulages; once developed, will there be chances to re-use the moulage templates with other learners? Could cooperation with other health professions institutions reduce the cost for an individual site?

The relatively high (initial) effort required in the development of moulages calls for cost-benefit analysis [27]. The evidence available so far seems limited. In the wake of a systematic review investigating the impact of moulages on learner engagement, Stokes-Parish and colleagues [28] found only a small body of literature on moulages in simulation, with most only investigating the field of dermatology. Several studies were poorly reported and yielded only low-level evidence, so that the sum of generalizable findings on moulages in simulation is still undersized.

Two studies made use of moulages to have students experience the impact that dermatological diagnoses have on patients (psoriasis [16] and melanoma [29]). While the effects on students were remarkable, this also raises the question if SPs as wearers of such moulages need special debriefing and support.

### Strengths and limitations

In our article, we describe methods for developing and producing 2-dimensional moulages. This description should be accurate enough for others to copy the process and adapt it to their needs locally. We believe that it is important to share this knowledge in stark contrast, for example, to the historical moulage artists, who usually kept their recipes secret throughout history [30].

For development, we worked very closely with clinical experts. This not only benefits moulage authenticity but also increases their acceptance, since the experts were involved right from the start. To date our team developed and set up a manufacturing process. In the future, more exchange and cooperation with other simulation educators would be desirable. Our approach to evaluation of the moulages has so far been predominantly qualitative. In the future, this could also be done in a quantitative manner, e.g. using the MARS instrument [25] and include their effect in simulations [2].

### Using 2D transfer tattoos on different skin tones

There seems to be a bias in dermatology education (but not restricted to that discipline) that is attracting increasing attention. It has been shown that educational materials underrepresent patients with darker skin tones [31] e.g., in dermatology textbooks [32]. Along this line, it appears that most academic work on modern medical moulage also stems from North America and Europe. This might well be another aspect of that bias that needs to be addressed.

The authors’ experiences stem from a Central European simulation and SP program that, while actively working on its diversity, so far has most experience with moulage on light-skinned SP.

The production of printed transfer tattoos as described in this report utilizes a colour pigment on a quasi-transparent film that is transferred to the SP’s own skin surface. From a technological standpoint, the challenge lies in a basic conception of inkjet printers: common printers mix five colours to achieve the desired print, CMYK and white, with the latter not added from a cartridge but contributed by the white printer paper. However, when printing moulage, the fifth colour in the mix is the SP’s own skin colour, which now limits the range of printable findings. This effect is less prominent in lighter skin tones that have a higher white fraction than in darker skin tones. One approach to counter this limitation would be the use either of white printer toner, or, adding a second, partially opaque white base film below the top colour layer that depicts the pathology, but both approaches warrant further experiments. From an integrative standpoint on the other side, this calls for a closer look how the preparation and use of moulage might differ for SP with different skins tones. The fact that moulage have been successfully used e.g. in Brazil, South Africa, Pakistan, or Spain, where darker skin tones are more common [11, 33–35], as well as settings with more diverse SP [14] shows that the concept of moulage in education is as such not restricted by skin tones.

## Conclusions

The use of modern medical moulage can enhance simulation-based education and assessment. Moulages offer the possibility to advance simulation of a wide range of pathologies and conditions. Due to their high authenticity and standardization, 2-dimensional transfer tattoos are ideal for use in high-stakes assessment. Since the range of commercially available moulages is narrow and their authenticity not always guaranteed we decided to share our experiences in this report. By doing so, we hope health profession educators can overcome the obstacles in moulage preparation and successfully and independently produce their own that best fit their local educational needs.

## Supplementary Information

**Additional file 1.** “MakeUp_DB_hohe.Quali.mp4”. The video file shows the transfer of a 2-dimensional print onto an SP.

## Data Availability

Not applicable.

## Footnotes

1 Colour fidelity is influenced by several factors. Full colour management can prove quite resource-consuming: Each parameter would need its own ICC profile, which can only be achieved by graphics professionals carrying out the calibration. Further, printing/scanning equipment at a given institution cannot be changed easily. Last but not least, the final output medium, i.e. the SP’s skin, cannot be covered technically (visible edges), or standardized at that, why full colour management remains unfeasibly after all. Considering this, we suggest for an iterative opportune colour management approach with partially calibrated devices, visual image editing using standard profiles, and multiple proofs to ensure flexibility with limited resources. In the course of an opportune colour management, we have also renounced the ICC profiling of the printer. Instead, the printer should be located in close proximity to image processing.

2 When using sealer that has to be mixed beforehand, prepare in time and according to manufacturer’s specifications. We recommend Bluebird FX sealer (matt or gloss), mixed with 96% isopropyl at a 1:1 ratio.

3 In cases where some strain on the tattoo is foreseeable, apply the tattoo, let it dry, apply first layer of sealer, let it dry, then apply second layer of sealer and again leave to dry.

4 Although the application can be done by lay people, we still recommend training in the application and post-production process.
